# H7N6 highly pathogenic avian influenza in Mozambique, 2023

**DOI:** 10.1080/22221751.2024.2321993

**Published:** 2024-02-29

**Authors:** Iolanda Vieira Anahory Monjane, Hernâni Djedje, Esmeralda Tamele, Virgínia Nhabomba, Almiro Rogério Tivane, Zacarias Elias Massicame, Dercília Mudanisse Arone, Ambra Pastori, Alessio Bortolami, Isabella Monne, Timothy Woma, Charles E. Lamien, William G. Dundon

**Affiliations:** a Directorate of Animal Science, Central Veterinary Laboratory, Agrarian Research Institute of Mozambique, Maputo, Mozambique; b Mozambique One Health Secretariat, National Health Institute, Maputo, Mozambique; c Ministry of Agriculture and Rural Development, National Directorate of Livestock Development, Maputo, Mozambique; d Division of Comparative Biomedical Sciences (BSBIO), Istituto Zooprofilattico Sperimentale delle Venezie (IZSVe), Padova, Italy; e Emergency Centre for Transboundary Animal Diseases (ECTAD), Food and Agriculture Organization (FAO), Maputo, Mozambique; f Animal Production and Health Laboratory, IAEA Laboratories, Seibersdorf, Austria

**Keywords:** Highly pathogenic avian influenza, H7N6, Mozambique, poultry, outbreak

## Abstract

On 13 October 2023, the National Directorate for Livestock Development in Mozambique was notified of a suspected outbreak of avian influenza in commercial layers. Samples were screened by real-time and conventional RT–PCR and were positive for both H7 and N6. Full genome sequences were obtained for three representative samples. Sequence analysis of the H7 cleavage site confirmed that the viruses were highly pathogenic (i.e. 333- PEPPKGPRFRR/GLF-346). In addition, the H7 and N6 sequences were highly similar (from 99.4-99.5% and 99.6-99.7% for the HA gene and the NA gene, respectively) to the sequences of a H7N6 virus identified in the Republic of South Africa in May 2023 indicating a similar origin of the viruses. The identification of H7N6 HPAIV in Mozambique has important implications for disease management and food security in the region.

Highly pathogenic avian influenza (HPAI) is caused by H5 and H7 subtypes of type A influenza virus. In the last number of years, global attention has been focused on the spread and impact of H5Nx viruses that have caused devastating outbreaks in both domestic and wild bird populations worldwide [1]. However, H7 subtype viruses have also caused numerous outbreaks in domestic poultry. Examples include H7N1 outbreaks in Italy; H7N2 outbreaks in Australia, USA, UK; H7N3 in Canada, Chile, Italy, Mexico, Pakistan; H7N4 in Australia; H7N7 in Australia, Germany, Italy, Spain, the Netherlands, United Kingdom; H7N8 and H7N9 in the USA [2, 3].

The most recent outbreaks caused by an H7 subtype virus were reported in the Republic of South Africa (RSA) in May 2023 [4]. This H7N6 virus has resulted in the death or culling of tens of thousands of poultry (layers). Following the occurrence of the H7N6 (and previous H5N8, H5N1, H5N2 outbreaks) in the RSA, the Ministry of Agriculture and Rural Development (MADER) in Mozambique suspended the importation of live domestic and wild birds and poultry products from the RSA on 6 October 2023. Additionally, prevention and surveillance measures were reinforced throughout the national territory.

As a result of the intensification of these measures, on 13 October 2023, MADER was notified of the suspected occurrence of avian influenza in a commercial poultry farm in the district of Morrumbene, Inhambane province, 500 km northeast of the capital Maputo. The birds showed enteric and respiratory symptoms with increased morbidity and mortality (the mortality was an average of 400 birds per day). The farm consisted of three sheds housing 45,000 birds (approximately 15,000 thousands birds perished). Abnormal mortality was recorded by the farm on September 25th with a total of 64 dead birds. On September 29th, the company reported the death of 102 birds in shed #1. On October 5th, the two remaining sheds recorded unusual mortality with cases increasing in shed #2 from 14 to 28 and in shed #3 from 12 to 57. Two days later the number of dead birds increased dramatically to 128 in shed #2 and 214 in shed #3. Virus spread was believed to have been due to poor biosecurity and the movement of individuals between the sheds. It was also determined that 45,000 live birds (17 weeks of age) were introduced into the farm between 30 August and 7 September 2023 in three different batches of 15,000 birds per batch, from an establishment certified as free of avian influenza in the North West province of the RSA.

In view of the outbreak situation, the Mozambican authorities implemented a number of measures which included movement restriction of poultry and poultry products from the Morrumbene district, sequestration and culling of all birds in the affected farm, destruction of feed (947 50 kg bags), waste, poultry litter and other material used or generated by poultry production, collection and destruction of eggs (approximately 88,000) distributed and/or sold after September 15th. Disinfection of infected equipment and sheds with Superquat (1:200) (Deluxe Chemicals, RSA) was undertaken after the culling of the birds.

Dead birds (*n* = 10) with suspected AI were sent to the Central Veterinary Laboratory (CVL) in Maputo. At the CVL, necropsy was performed and trachea (*n* = 10), lung (*n* = 10) samples were collected and pooled for molecular testing and outbreak confirmation (Table 1). Following necropsy, RNA was purified from the samples and screened for type A, H5 subtype and H7 subtype AIV using standard real-time RT-qPCR protocols [5, 6]. All of the samples were positive for type A and H7 and were negative for H5 AIV. The outbreak was officially report to the World Oganization for Animal Health (WOAH) on the 17th of October 2023 [7]. No further outbreaks have been reported since.

**Table 1.** Samples anlaysed in this study.

#	Sample code	Host	Collection date	Samples type	H7N6	Genome
1	855P4-1	Chicken layer	16-10-2023	Pooled trachea/lung	+	+
2	855P4-2	Chicken layer	16-10-2023	Pooled trachea/lung	+	-
3	855P4-3	Chicken layer	16-10-2023	Pooled trachea/lung	+	-
4	855P4-4	Chicken layer	16-10-2023	Pooled trachea/lung	+	-
5	856P5-1	Chicken layer	16-10-2023	Pooled trachea/lung	+	-
6	856P5-2	Chicken layer	16-10-2023	Pooled trachea/lung	+	-
7	856P5-3	Chicken layer	16-10-2023	Pooled trachea/lung	+	-
8	856P5-4	Chicken layer	16-10-2023	Pooled trachea/lung	+	+
9	857P3-1	Chicken layer	16-10-2023	Pooled trachea/lung	+	+
10	857P3-2	Chicken layer	16-10-2023	Pooled trachea/lung	+	-

The same swab, lung and trachea samples were sent to the WOAH/Food and Agriculture Organization/European Union Reference Laboratory for Avian Influenza and Newcastle Disease, Legnaro, Italy for diagnostic confirmation and genetic characterization (see supplementary material and methods). All of the samples were confirmed to be positive for H7N6 and the full genome of three viruses (i.e. A/chicken/Mozambique/857-P3-1_23VIR11699-9/2023, A/chicken/Mozambique/855-P4-1_23VIR11699-1/2023 and A/chicken/Mozambique/855-P5-4_23VIR11699-8/2023) from representative samples were generated (see supplementary material and methods). Sequences of the eight gene segments were submitted to GenBank (accession numbers PP112211 to PP112218, PP112222 to PP112237) and compared with the most related virus sequences available in GISAID, selected based on a BLAST search. Maximum likelihood phylogenetic trees were generated using IQTree v.1.6.12. (see supplementary material and methods). Phylogenetic analyses of the HA and NA genes (Figure 1A and B) show that the three HPAI H7N6 viruses from Mozambique cluster together and with an HPAI H7N6 virus collected from layers in Delmas, Mpumalanga, RSA at the end of May 2023 [A/chicken/South Africa/SA2310/2023 (H7N6)], for which only the HA and NA are available in GISAID (www.gisaid.org; accession numbers EPI2699047 and EPI2699049). Specifically, the nucleotide identity between the H7N6 viruses from Mozambique and the RSA ranged from 99.4-99.5% for the HA gene and from 99.6-99.7% for the NA gene. Topology of the phylogenetic trees obtained for the other gene segments (see supplementary Fig. S1) indicates that the H7N6 viruses from Mozambique are separated from the sequences of the other viruses by long branches, suggesting a gap in sequencing data. Based on the data publicly available, the PB2, PB1, NP and NS gene segments of the viruses under investigation cluster with low pathogenic avian influenza viruses of different subtypes collected from wild and domestic birds in the RSA and Zambia while, the PA and M gene segments cluster with European viruses. Figure 1.Maximum likelihood phylogenetic tree of the (A) HA segment and (B) NA segment obtained with IQtree 1.6.6. Ultrafast bootstrap values higher than 80 are indicated next to the nodes. Viruses from Mozambique are shown in red.

**Figure:**
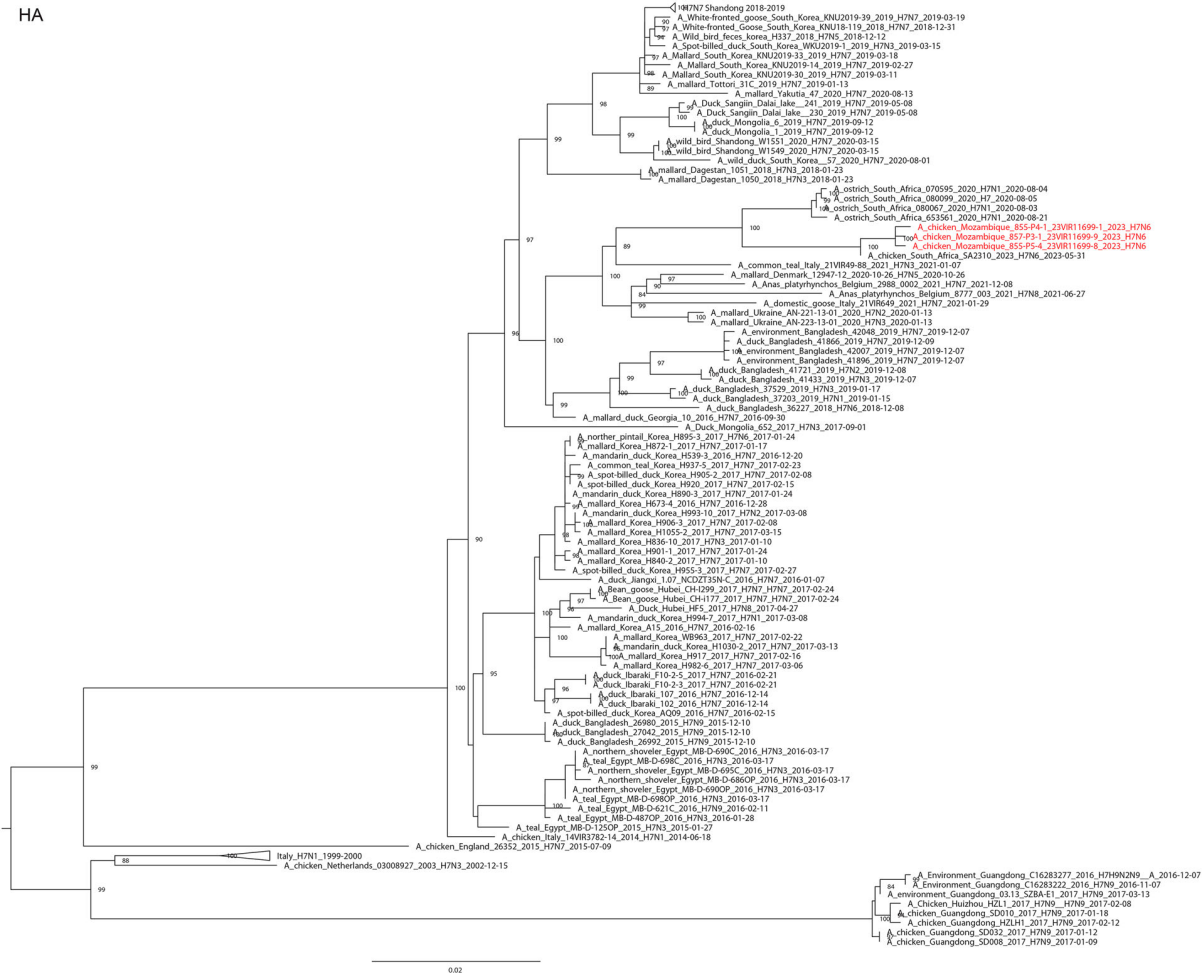

**Figure:**
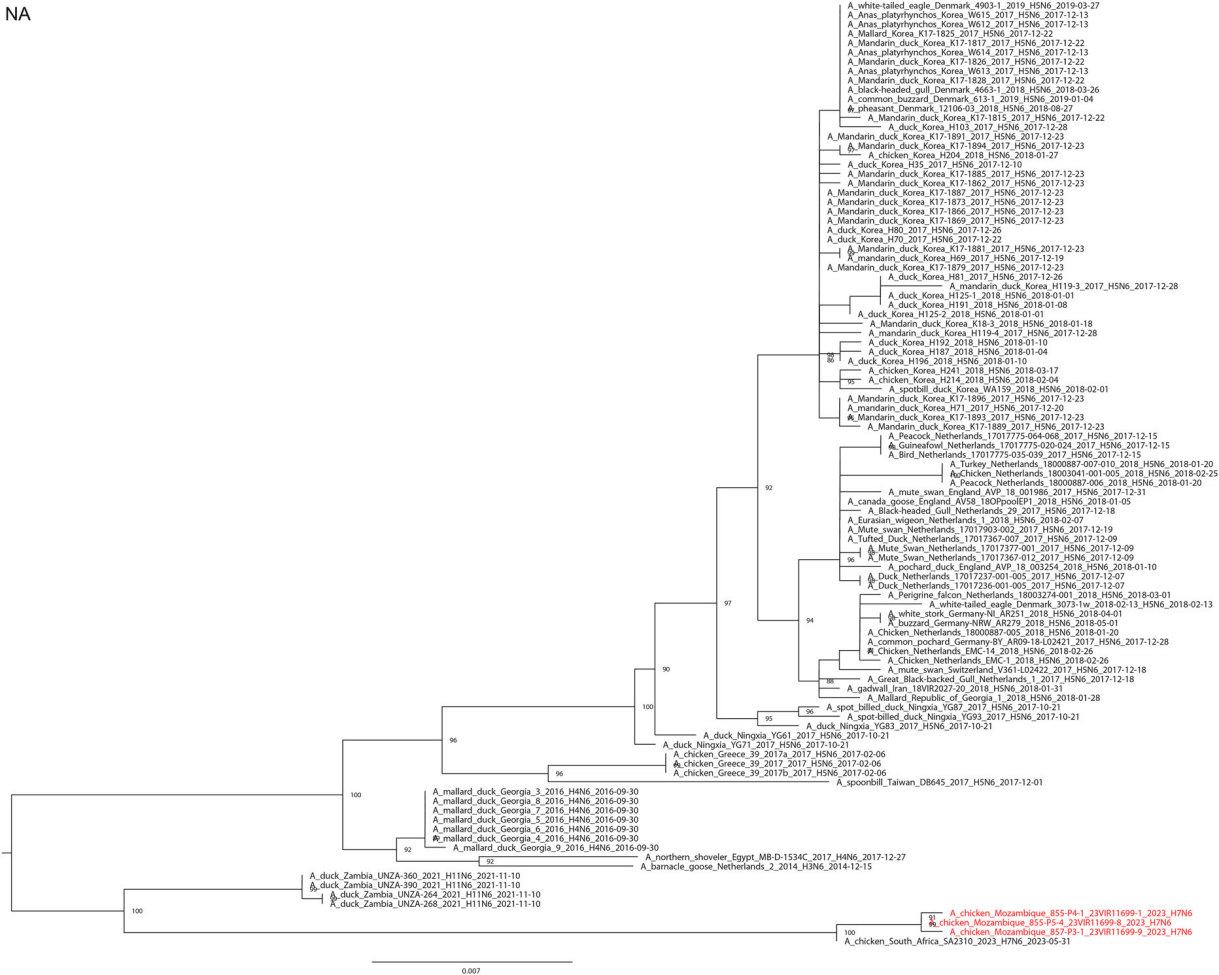

Sequence analysis of the H7 segment identified a cleavage site (333- PEPPKGPRFRR/GLF-346) characteristic of HPAIs. The same cleavage site was present in the H7N6 virus from the RSA A/chicken/South Africa/SA2310/2023. Compared to the H7N6 virus from the RSA, no additional glycosylation sites were observed in the HA and NA proteins. Additionally, mutations associated with virus adaptation in mammals were identified in the viral proteins of the analysed viruses and are listed in supplementary Table S1. The effect of these mutations on the biological characteristics of these viruses is unknown and further studies are required.

The zoonotic potential of this new virus should be considered. H7N2, H7N3, H7N7 viruses have all been previously associated with conjunctivitis and/or respiratory symptoms in humans while in 2003 in the Netherlands a fatal case occurred following an infection with H7N7 [8, 9]. In March 2013, cases of human avian influenza A(H7N9) were reported in China and to date there have been 616 recorded deaths due to this virus [10]. More recently, in 2018, a human infection by an H7N4 virus was reported in China [11].

To date, there have been several identifications of H7N6 subtype viruses in wild birds but they have all been of low pathogenicity. There have been some recent reports of low pathogenic H7N6 viruses in poultry in Chile and Cambodia but none of these viruses have caused significant disease [12, 13].

Currently, there is not enough data publicly available on the circulation of H7N6 in the southern African region to make meaningful comparisons and to identify a definitive source of the outbreak in Mozambique but there is no doubt that there is a molecular epidemiological link between the H7N6 viruses identified in Mozambique and those in the RSA. Analysis of viruses from similar outbreaks in the region will add greatly to understanding the movement and potential impact of this new subtype. In the meantime, a risk based active surveillance should be initiated with national veterinary authorities remaining vigilant and prepared to control and manage possible future outbreaks of H7N6.

## Supplementary Material

Supplementary_material

Figure_S1
