# Supplementary material for: H7N6 highly pathogenic avian influenza in Mozambique, 2023
Source: Emerg Microbes Infect. 2024 Feb 29;13(1):2321993. doi: 10.1080/22221751.2024.2321993 (PMC10906114; doi:10.1080/22221751.2024.2321993)
Supplement: Supplementary_material [file TEMI_A_2321993_SM6727.docx]

**Monjane et al**: **H7N6 highly pathogenic avian influenza in Mozambique, 2023**

**Supplementary material and methods**

**Virus identification**

All the M-gene positive samples were tested for H7 subtype through rRT-PCR using a protocol based on the assay developed by Van Borm et al. [1], adopting the probe as modified by Hoffmann et al. [2]. A one-step RT-PCR protocol was used in order to identify the pathotype via Sanger sequencing of the hemagglutinin cleavage site [3]. Neuraminidase subtyping (N6) was performed through rRT-PCR as previously described [4].

**Genome sequencing**

A target RT-PCR approach was used to amplify influenza A virus whole genomes from three HPAI H7N6 positive clinical specimens as previously described [5]. Sequencing libraries were obtained using the Illumina DNA prep kit (Illumina, San Diego, CA, USA) and sequenced on MiSeq instrument using a 2x 250 PE mode. Raw sequencing reads produced by MiSeq instrument were cleaned with Trimmomatic v0.32 with minimum quality 20. Illumina DNA prep adapter sequences were clipped from reads using scythe v0.991 (https://github.com/vsbuffalo/scythe) and amplification primers were removed using sickle v1.33 (https://github.com/najoshi/sickle). Read shorter than 80bp or unpaired were discarded. The cleaned reads were aligned against a reference genome using the MEM algorithm from BWA v0.7.12-r1039. Picard tools v2.1.0 (http://broadinstitute.github.io/picard/) and GATK v3.530-32 [6] were used to improve alignment quality, correct potential errors and recalibrate base quality score. LoFreq v2.1.2.33 [7] was used to call single nucleotide polymorphisms which were reported in a vcf file. The generated vcf file was then used to produce the consensus sequences using an in-house script. Briefly, this script calls the base for each position with a coverage >10X, considering all the polymorphysms with a frequency higher than 25%. “N” is assigned to all positions with a coverage lower than 10 reads.

**Phylogenetic analysis**

Consensus sequences were aligned using MAFFT v7 online server <https://mafft.cbrc.jp/alignment/server/>) [8] and compared with sequences of most related virus strains available in GISAID. Maximum likelihood phylogenetic trees of each gene segment were obtained by using IQTREE v1.6.6 (https://github.com/iqtree/iqtree1) and its robustness was determined through an ultrafast bootstrap resampling analysis of 1000 replications [9]. Phylogenetic trees were visualized using Fig Tree v1.4.4 software (<http://tree.bio.ed.ac.uk/software/figtree/>).

**Table S1.** Molecular markers identified in the analysed viruses.

| **Marker** | **Effects** | **Reference** |
| --- | --- | --- |
| PB2:K389R | Increased polymerase activity in mammalian cells; Increased replication in mammalian cells | [10, 11] |
| PB2:V598T | Increased virulence in mice; Increased polymerase activity in mammalian cells; Increased replication in mammalian cells | [10, 11] |
| PB2:I292V | Increased virulence in mice; Increased polymerase activity in mammalian cells | [11-13] |
| PB2:L89V;PB2:G309D | Increased polymerase activity in mammalian cells; Increased virulence in mice | [11, 14] |
| PB1-F2:N66S | Enhanced replication in mice; Enhanced virulence in mice | [11,15,16] |
| PB1:D622G | Increased polymerase activity in mice; Increased virulence in mice | [11] |
| PA:S37A | Increased polymerase activity in mammalian cells | [11, 17] |
| PA:P190S | Decreased virulence in mice | [11, 18] |
| PA:N409S | Increased polymerase activity in mammalian cells; Increased replication in mammalian cells | [11, 17] |
| PA:V63I | Increased polymerase activity in mammalian cells; Enhanced replication in mammalian cells; Increased virulence in mice | [10, 11, 19] |
| M1:N30D | Increased virulence in mice | [11, 20] |
| M1:T215A | Increased virulence in mice | [11, 20] |
| NS1:I106M | Increased viral replication in mammalian cells; Increased virulence in mice | [11, 21] |
| NS1:C138F | Increased viral replication in mammalian cells; Decreased interferon response | [11] |

**References**

1. Van Borm S, Suarez DL, Boschmans M, Ozhelvaci O, Marché S, van den Berg TP. Rapid detection of Eurasian and American H7 subtype influenza A viruses using a single TaqManMGB real-time RT-PCR. Avian Dis. 2010 Mar;54(1 Suppl):632-8. doi: 10.1637/8734-032509-ResNote.1.
2. Hoffmann B, Hoffmann D, Henritzi D, Beer M, Harder TC. Riems influenza a typing array (RITA): An RT-qPCR-based low density array for subtyping avian and mammalian influenza a viruses. Sci Rep. 2016 Jun 3;6:27211. doi: 10.1038/srep27211.

Slomka MJ, Coward VJ, Banks J, Löndt BZ, Brown IH, Voermans J, Koch G, Handberg KJ, Jørgensen PH, Cherbonnel-Pansart M, Jestin V, Cattoli G, Capua I, Ejdersund A, Thorén P, Czifra G. Identification of sensitive and specific avian influenza polymerase chain reaction methods through blind ring trials organized in the European Union. Avian Dis. 2007 Mar;51(1 Suppl):227-34. doi: 10.1637/7674-063006R1.1.

1. James J, Slomka MJ, Reid SM, Thomas SS, Mahmood S, Byrne AMP, Cooper J, Russell C, Mollett BC, Agyeman-Dua E, Essen S, Brown IH, Brookes SM. Proceedings Paper-Avian Diseases 10th AI Symposium Issue Development and Application of Real-Time PCR Assays for Specific Detection of Contemporary Avian Influenza Virus Subtypes N5, N6, N7, N8, and N9. Avian Dis. 2019 Mar 1;63(sp1):209-218. doi: 10.1637/11900-051518-Reg.1.
2. Fusaro A, Zecchin B, Vrancken, B, et al. Disentangling the Role of Africa in the Global Spread of H5 Highly Pathogenic Avian Influenza. *Nat. Commun.* 2019; *10*, doi:10.1038/S41467-019-13287-Y.
3. Van der Auwera GA, Carneiro MO, Hartl C, et al. From FastQ Data to High Confidence Variant Calls: The Genome Analysis Toolkit Best Practices Pipeline. Curr. Protoc. Bioinformatics 2013;(43): 11.10.1-33, doi:10.1002/0471250953.bi1110s43.
4. Wilm A, Aw PPK, Bertrand D, et al. A Sequence-Quality Aware, Ultra-Sensitive Variant Caller for Uncovering Cell-Population Heterogeneity from High-Throughput Sequencing Datasets. Nucleic Acids Res. 2012; (40): 11189–11201, doi:10.1093/nar/gks91
5. Katoh K, Standley DM. MAFFT Multiple Sequence Alignment Software Version 7: Improvements in Performance and Usability. Mol. Biol. Evol. 2013, (30): 772–780, doi:10.1093/MOLBEV/MST010.
6. Hoang DT, Chernomor O, Von Haeseler A, UFBoot2: Improving the Ultrafast Bootstrap Approximation. Mol. Biol. Evol. 2018;(35): 518–522, doi:10.1093/MOLBEV/MSX281.
7. Hu M, Yuan S, Zhang K, Singh K, Ma Q, Zhou J, Chu H, Zheng BJ. PB2 substitutions V598T/I increase the virulence of H7N9 influenza A virus in mammals. Virology. 2017 Jan 15;501:92-101. doi: 10.1016/j.virol.2016.11.008.
8. Suttie A, Deng YM, Greenhill AR, Dussart P, Horwood PF, Karlsson EA. Inventory of molecular markers affecting biological characteristics of avian influenza A viruses. Virus Genes. 2019 Dec;55(6):739-768. doi: 10.1007/s11262-019-01700-z.
9. Xiao C, Ma W, Sun N, Huang L, Li Y, Zeng Z, Wen Y, Zhang Z, Li H, Li Q, Yu Y, Zheng Y, Liu S, Hu P, Zhang X, Ning Z, Qi W, Liao M. PB2-588 V promotes the mammalian adaptation of H10N8, H7N9 and H9N2 avian influenza viruses. Sci Rep. 2016 Jan 19;6:19474. doi: 10.1038/srep19474.
10. Gao W, Zu Z, Liu J, Song J, Wang X, Wang C, Liu L, Tong Q, Wang M, Sun H, Sun Y, Liu J, Chang KC, Pu J. Prevailing I292V PB2 mutation in avian influenza H9N2 virus increases viral polymerase function and attenuates IFN-β induction in human cells. J Gen Virol. 2019 Sep;100(9):1273-1281. doi: 10.1099/jgv.0.001294.
11. Li J, Ishaq M, Prudence M, Xi X, Hu T, Liu Q, Guo D. Single mutation at the amino acid position 627 of PB2 that leads to increased virulence of an H5N1 avian influenza virus during adaptation in mice can be compensated by multiple mutations at other sites of PB2. Virus Res. 2009 Sep;144(1-2):123-9. doi: 10.1016/j.virusres.2009.04.008.
12. Conenello GM, Zamarin D, Perrone LA, Tumpey T, Palese P. A single mutation in the PB1-F2 of H5N1 (HK/97) and 1918 influenza A viruses contributes to increased virulence. PLoS Pathog. 2007 Oct 5;3(10):1414-21. doi: 10.1371/journal.ppat.0030141.
13. Schmolke M, Manicassamy B, Pena L, Sutton T, Hai R, Varga ZT, Hale BG, Steel J, Pérez DR, García-Sastre A. Differential contribution of PB1-F2 to the virulence of highly pathogenic H5N1 influenza A virus in mammalian and avian species. PLoS Pathog. 2011 Aug;7(8):e1002186. doi: 10.1371/journal.ppat.1002186.
14. Yamayoshi S, Yamada S, Fukuyama S, Murakami S, Zhao D, Uraki R, Watanabe T, Tomita Y, Macken C, Neumann G, Kawaoka Y. Virulence-affecting amino acid changes in the PA protein of H7N9 influenza A viruses. J Virol. 2014 Mar;88(6):3127-34. doi: 10.1128/JVI.03155-13.
15. DesRochers BL, Chen RE, Gounder AP, Pinto AK, Bricker T, Linton CN, Rogers CD, Williams GD, Webby RJ, Boon AC. Residues in the PB2 and PA genes contribute to the pathogenicity of avian H7N3 influenza A virus in DBA/2 mice. Virology. 2016 Jul;494:89-99. doi:10.1016/j.virol.2016.04.013.
16. Hu M, Chu H, Zhang K, Singh K, Li C, Yuan S, Chow BK, Song W, Zhou J, Zheng BJ. Amino acid substitutions V63I or A37S/I61T/V63I/V100A in the PA N-terminal domain increase the virulence of H7N7 influenza A virus. Sci Rep. 2016 Nov 25;6:37800. doi: 10.1038/srep37800.
17. Fan S, Deng G, Song J, Tian G, Suo Y, Jiang Y, Guan Y, Bu Z, Kawaoka Y, Chen H. Two amino acid residues in the matrix protein M1 contribute to the virulence difference of H5N1 avian influenza viruses in mice. Virology. 2009 Feb 5;384(1):28-32. doi: 10.1016/j.virol.2008.11.044.
18. Ayllon J, Domingues P, Rajsbaum R, Miorin L, Schmolke M, Hale BG, García-Sastre A. A single amino acid substitution in the novel H7N9 influenza A virus NS1 protein increases CPSF30 binding and virulence. J Virol. 2014 Oct;88(20):12146-51. doi: 10.1128/JVI.01567-14.
