# Supplementary figures and images for: H7N6 highly pathogenic avian influenza in Mozambique, 2023
Source: Emerg Microbes Infect. 2024 Feb 29;13(1):2321993. doi: 10.1080/22221751.2024.2321993 (PMC10906114; doi:10.1080/22221751.2024.2321993)

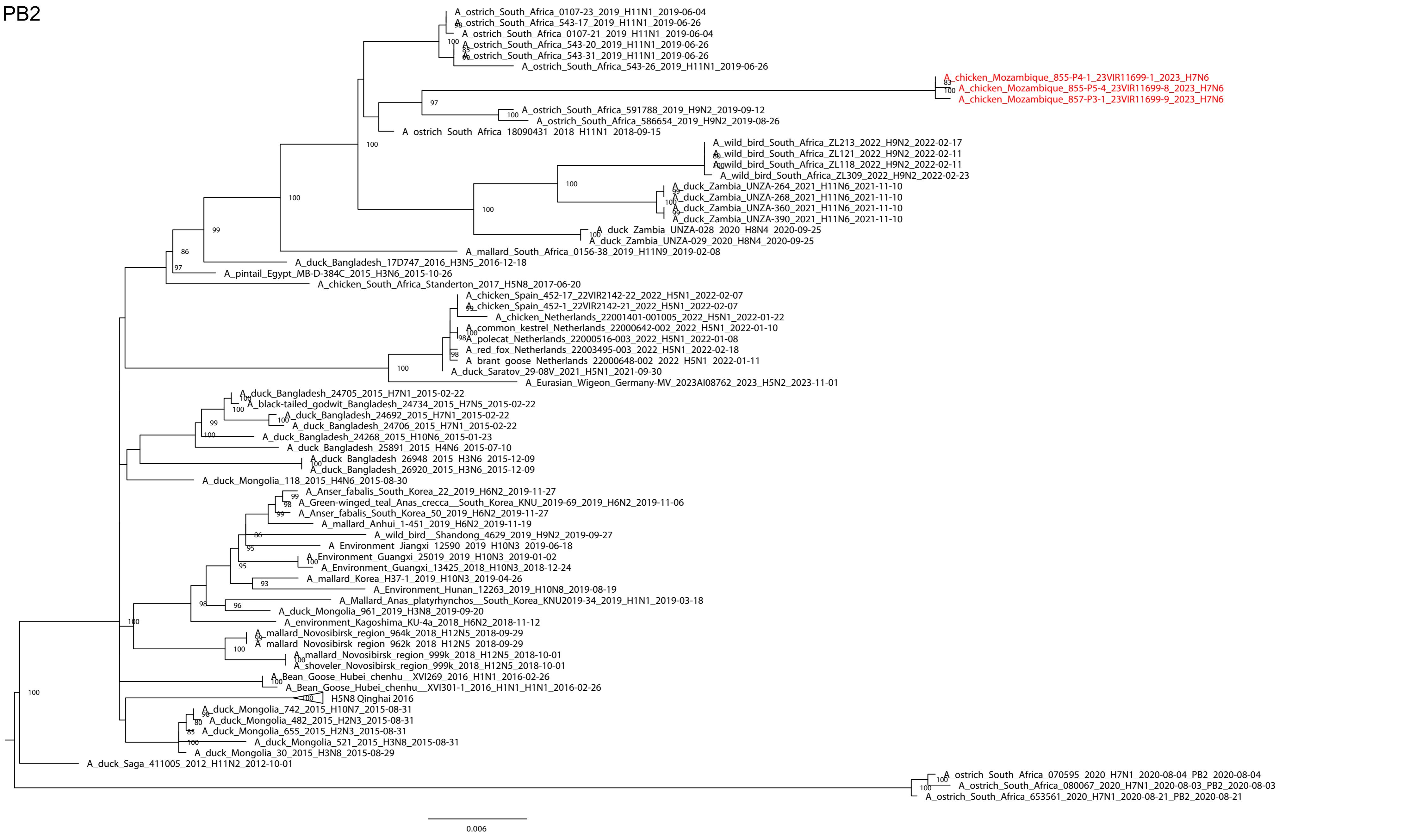

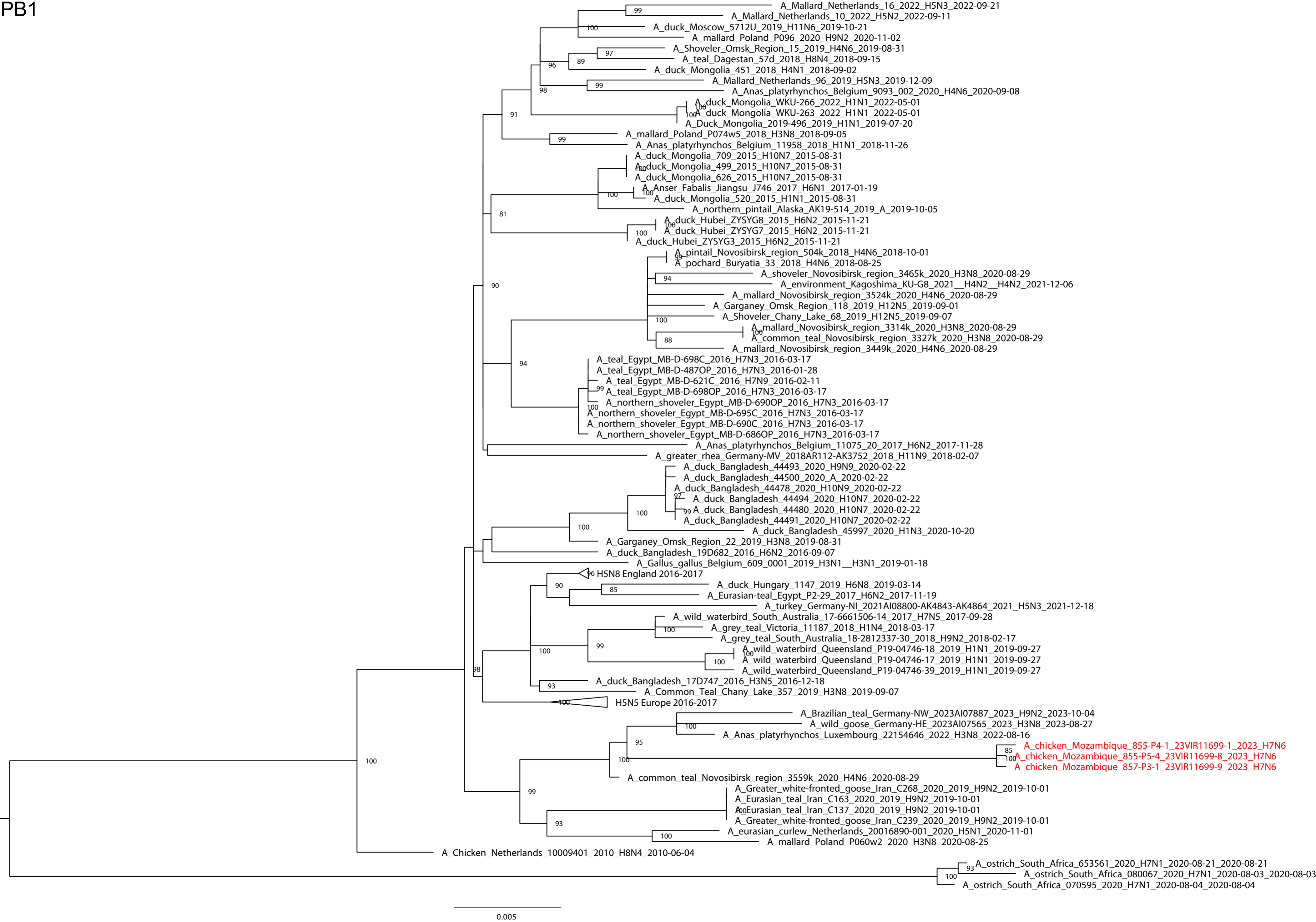

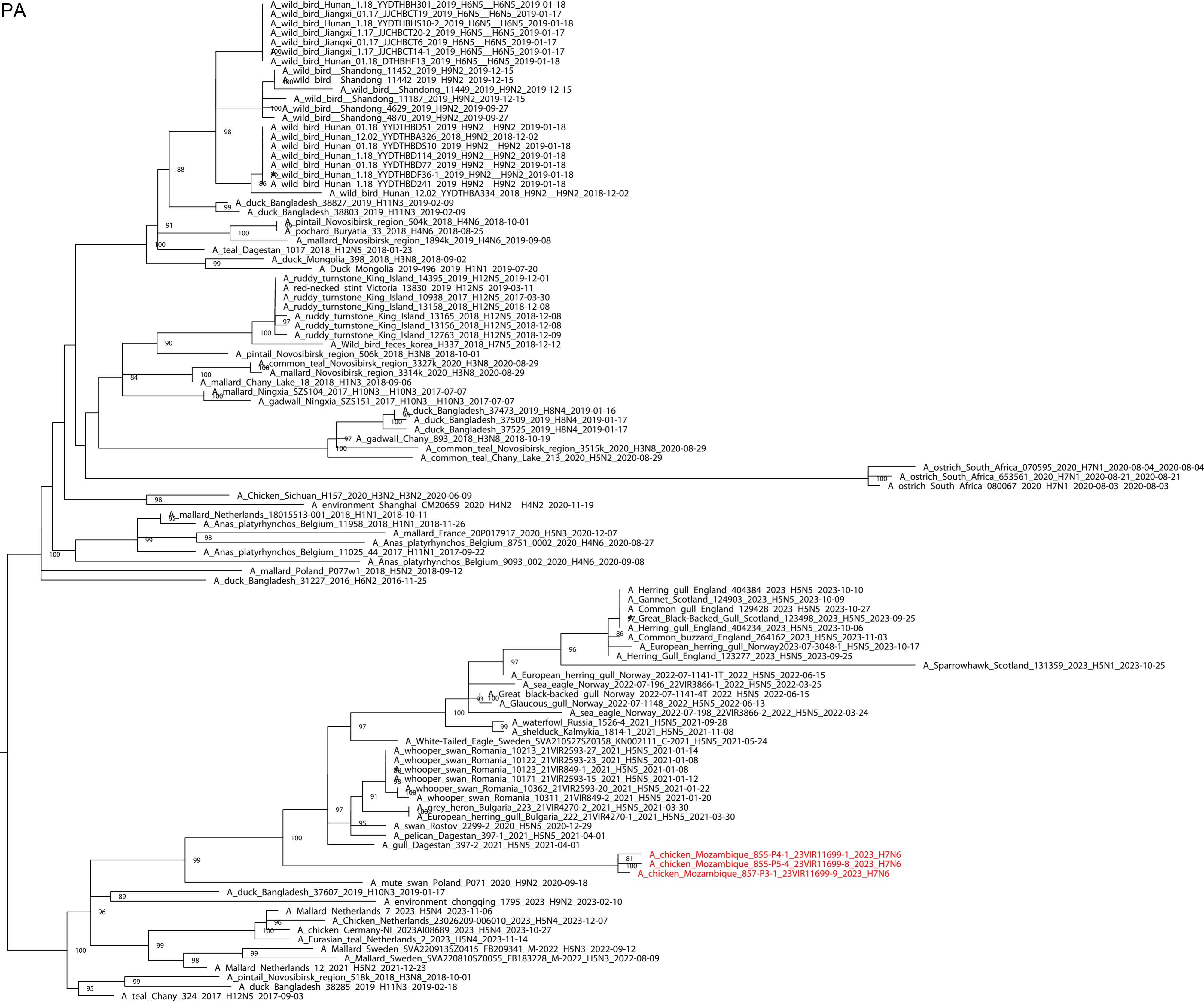

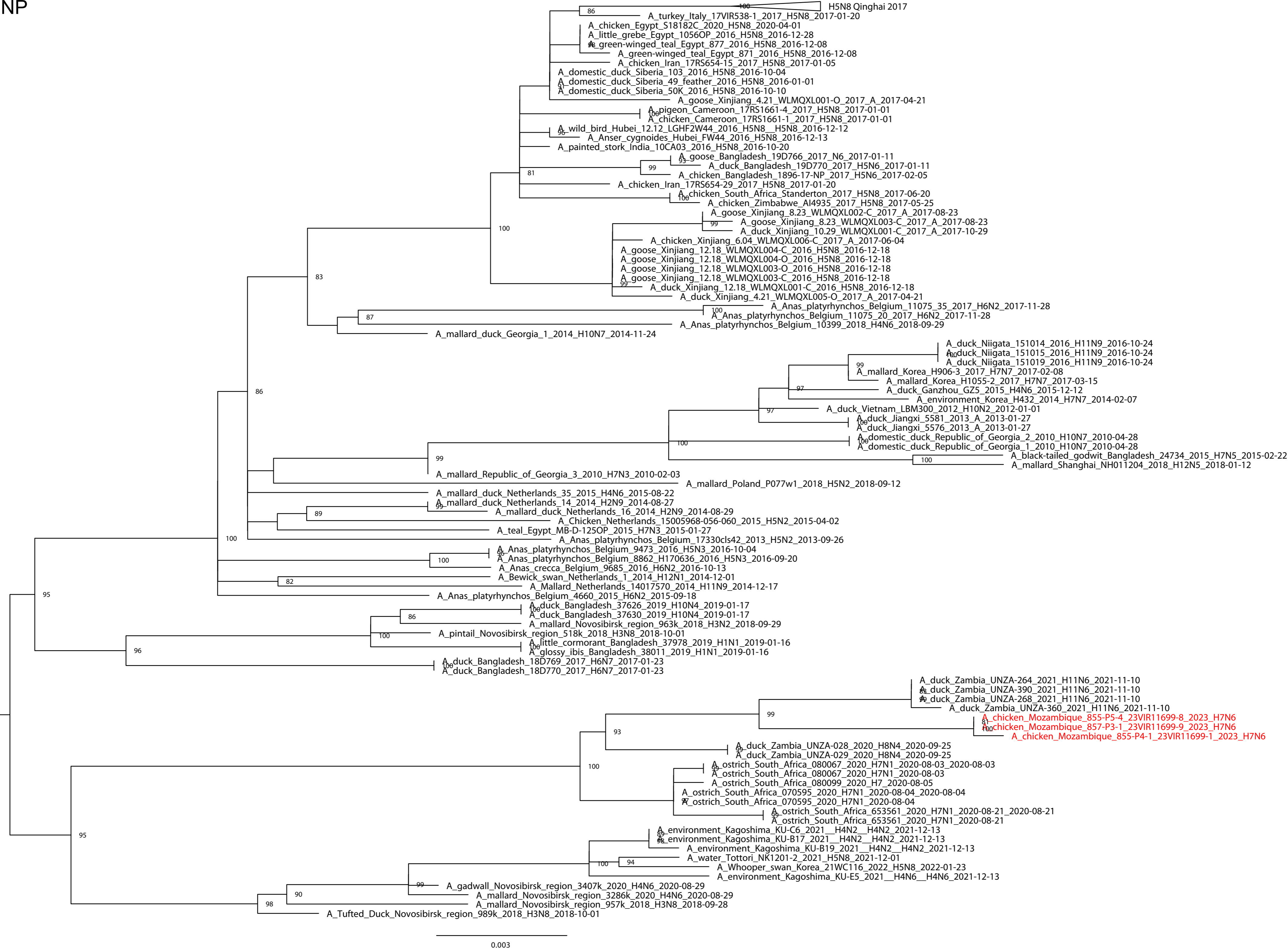

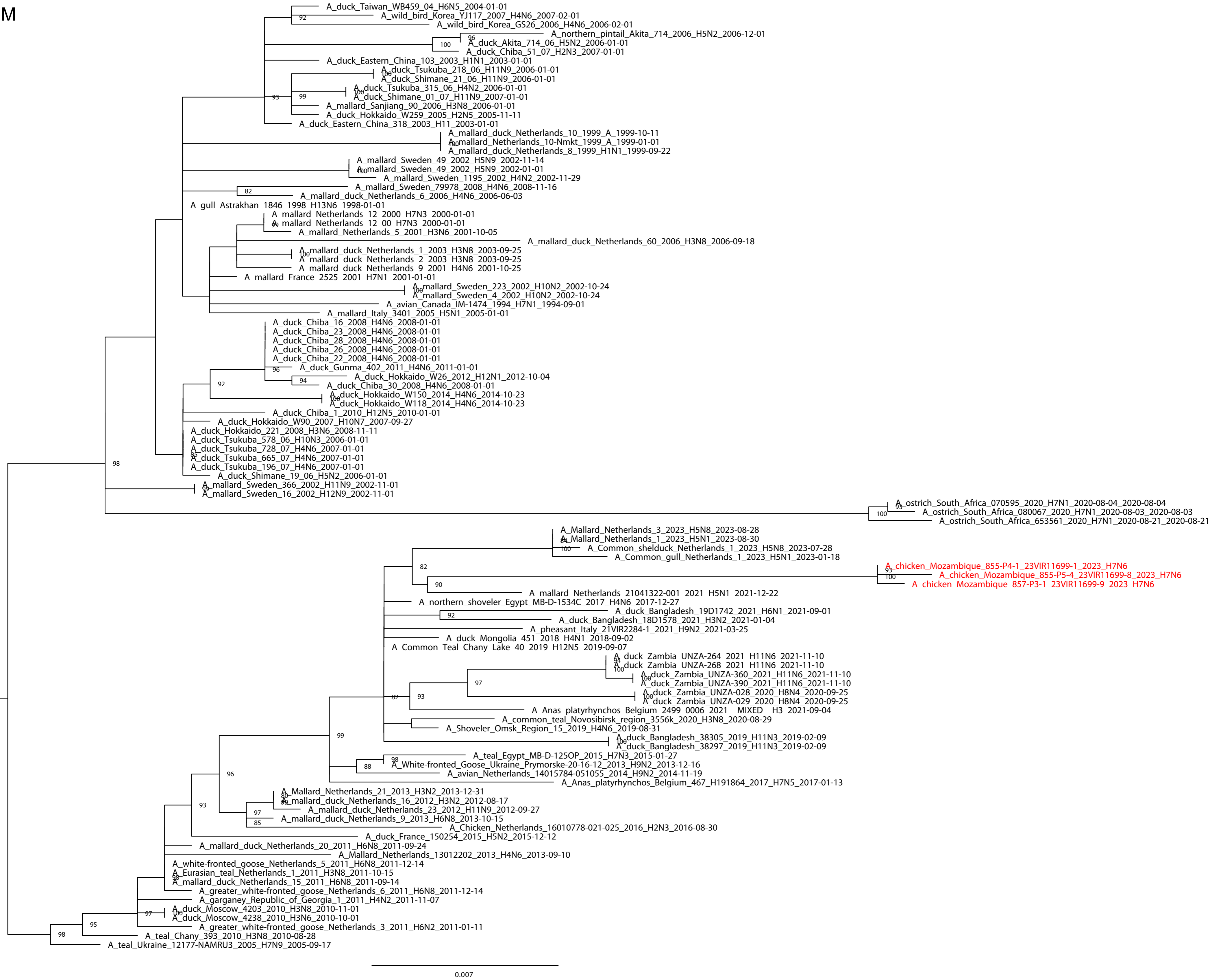

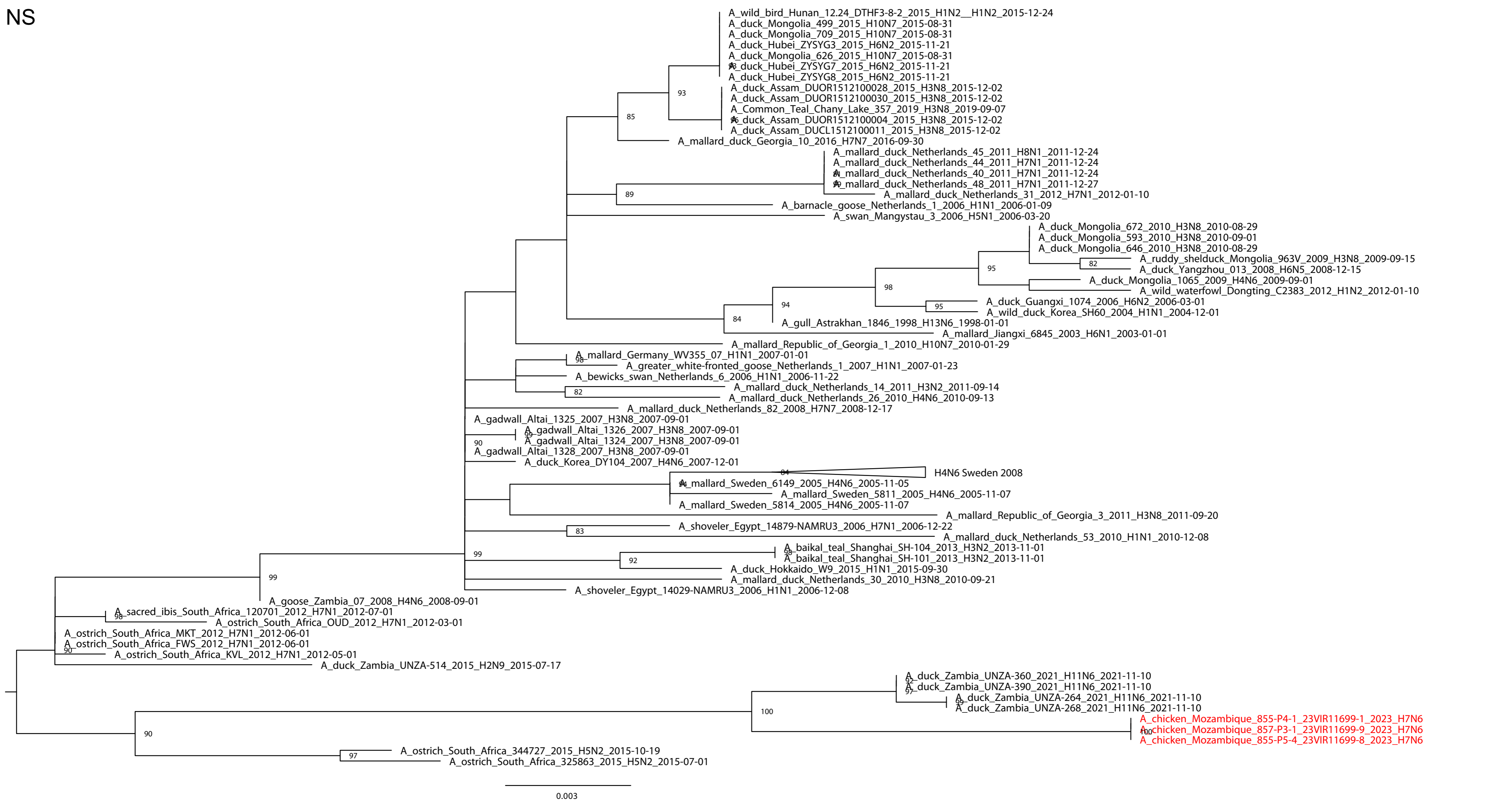

Supplement: Figure_S1 [file TEMI_A_2321993_SM6723.pdf]
